# Dynamic Hand Gesture Recognition Using Electrical Impedance Tomography

**DOI:** 10.3390/s22197185

**Published:** 2022-09-22

**Authors:** Xiuyan Li, Jianrui Sun, Qi Wang, Ronghua Zhang, Xiaojie Duan, Yukuan Sun, Jianming Wang

**Affiliations:** 1 School of Electronic and Information Engineering, Tiangong University, Tianjin 300387, China; 2 School of Artificial Intelligence, Tiangong University, Tianjin 300387, China; 3 School of Computer Science and Technology, Tiangong University, Tianjin 300387, China

**Keywords:** electrical impedance tomography (EIT), dynamic gesture recognition, excitation drive pattern, artificial intelligence, neural network

## Abstract

Electrical impedance tomography (EIT) has been applied in the field of human-computer interaction due to its advantages including the fact that it is non-invasive and has both low power consumption and a low cost. Previous work has focused on static gesture recognition based on EIT. Compared with static gestures, dynamic gestures are more informative and can achieve more functions in human-machine collaboration. In order to verify the feasibility of dynamic gesture recognition based on EIT, a traditional excitation drive pattern is optimized in this paper. The drive pattern of the fixed excitation electrode is tested for the first time to simplify the measurement process of the dynamic gesture. To improve the recognition accuracy of the dynamic gestures, a dual-channel feature extraction network combining a convolutional neural network (CNN) and gated recurrent unit (GRU), namely CG-SVM, is proposed. The new center distance loss is designed in order to simultaneously supervise the intra-class distance and inter-class distance. As a result, the discriminability of the confusing data is improved. With the new excitation drive pattern and classification network, the recognition accuracy of different interference data has increased by 2.7~14.2%. The new method has stronger robustness, and realizes the dynamic gesture recognition based on EIT for the first time.

## 1. Introduction

With continuous breakthroughs in methods and technologies, methods of human-computer interaction have been extensively enriched. Hand gesture recognition is one way to realize human-computer interaction by letting the computer recognize human gestures and the commands they represent. Hand gesture recognition can be divided into static hand gesture recognition and dynamic hand gesture recognition according to the recognized subject. Compared to static hand gesture recognition, dynamic hand gesture recognition has a more natural and comfortable interactive experience in some practical applications such as sign language recognition [1], interaction systems [2], virtual reality [3], interactive gaming [4], and human-machine collaborations [5].

In the past few decades, the recognition scheme of dynamic hand gestures mainly includes inertial motion sensing [6,7], electromyography (EMG) [8], and computer vision [9,10,11]. Inertial motion sensing system generally include accelerometers, gyro-scopes, and magnetometers. Faisal et al. [6] presented a sensor-based hand gesture recognition framework to classify both static and dynamic hand gestures in real-time using a data glove that contains a three-axis accelerometer, a three-axis gyroscope, and five flex sensors. However, the accelerometer sensor glove for gesture detection is large in size. The inertial motion sensor is limited to dynamic detection. Electromyography (EMG) can record bioelectrical signals generated by muscles through electronic instruments. This is an important method for noninvasive detection of muscle activity on the body’s surface. Qing et al. [8] studied gesture decoding based on surface EMG signals and discussed the effects of muscle fatigue, forearm angle, and acquisition time on the accuracy of gesture decoding. However, the electrodes of EMG are only used for receiving and conducting the electrical signal generated by muscle tissue. Thus, the signals they measured were very weak and vulnerable to noise. Computer vision is a technology that uses image system instead of visual organ as an input means and uses a computer instead of the brain to complete processing and interpretation. At present, the hand gesture recognition methods based on computer vision mainly include monocular visual recognition [9], binocular visual recognition [10], and depth visual recognition [11]. Although vision-based gesture recognition can distinguish between more complex hand postures, these systems have some significant limitations, including line-of-sight occlusion, ambient light noise, and higher computational costs. Wearable low-power gesture recognition methods have become the focus of attention.

Electrical impedance tomography (EIT) is a non-invasive imaging technology, which restores internal impedance distribution by measuring the response information of the surface electrode pair around the object [12]. In the medical field, it is mainly used for imaging of the chest, brain, and abdominal regions [13,14,15]. Since EIT has the advantages of being wearable and having both a low cost and low power consumption, it has proved effective for gesture recognition recently. During EIT data acquisition, electrode pairs at different positions are selected for excitation and measurement, which will produce different driving modes and measurement results. In previous studies, researchers explored and implemented different driving modes, such as adjacent driving mode, opposite driving mode, and cross driving mode, to verify the effect of excitation driving mode on image reconstruction quality. In the field of EIT gesture recognition, there are relatively few studies on the excitation driven pattern. Ma et al. [16] proposed a seven-electrode EIT drive pattern based on feature selection and model interpretation. The new pattern saves measurement time and reduces the number of electrodes used for gesture recognition. Jiang et al. [17] proposed an electrode layout for two wristbands. The 16 electrodes were evenly distributed on the two wristbands, which are placed at different positions of the arm. This layout is slightly better than the traditional single wristband since muscle impedance can be measured at two different locations. However, the existing excitation drive patterns for EIT gesture recognition are still based on traditional cyclic excitation and measurement method. Dynamic gesture recognition has attracted more and more attention due to its more informative and richer interactive functions. During the collection of dynamic gesture data, the arm muscles continue contracting. The periodic changes of the excitation position are apt to introduce measurement instability. In order to better respond to the impedance changes of dynamic gestures, a fixed excitation drive pattern was first attempted. Four fixed excitation measurement methods are discussed with respect to their ability to obtain an optimal excitation position.

Machine learning algorithms are not only applied to data prediction [18] and supervised learning [19], but have also been widely used to recognize gestures based on impedance data, such as K-nearest neighbor (KNN) [20], support vector machine (SVM) [21], decision tree [22], and so on. Zhang et al. [23] used support vector machine with a standard polynomial kernel to distinguish eight gestures. Yao et al. [24] tested three machine learning algorithms: radial basis kernel function (RBF) kernel support vector machine, decision tree, and quadratic discriminant classifier. They found that RBF support vector machine showed the best classification effect. Although traditional machine learning methods have fast training speeds in few-shot learning, their generalization ability is relatively weak. With deep learning theory and the improvement of numerical computing equipment, convolutional neural networks (CNN) have been developed rapidly. CNNs have powerful feature extraction capabilities and have been shown to achieve state-of-the-art performance in domains such as image classification, image segmentation, and object detection [25,26]. Leins et al. [27] used multi structured multilayer perceptron (MLP) networks and CNN networks to classify EIT gesture data, and they found that CNN networks have better stability and generalization abilities. However, CNNs are mainly used to extract spatial features of data and are not good at processing time related data with continuous features. In order to fully extract time series features from gesture data, a dual-channel feature extraction network combining a convolutional neural network and gated recurrent unit is proposed.

The contributions of our paper are threefold. Firstly, the most suitable EIT excitation driving pattern for dynamic gesture recognition is proposed. Secondly, the CG-SVM network is designed to improve anti-interference ability of gesture classification. Finally, the loss function of the classification network is improved to better distinguish the confusing gesture features.

The rest of the paper is organized as follows. Section 2 presents the design and implementation of the overall system architecture and details our improvement plans for the excitation drive pattern. Section 3 introduces the network structure of CG-SVM and the design principle of the center distance loss. The comparative experiments of different excitation drive pattern and various network classification results are discussed in Section 4. Concluding remarks are provided in Section 5.

## 2. System Implementation

### 2.1. Measurement Scheme

**Figure 1:**
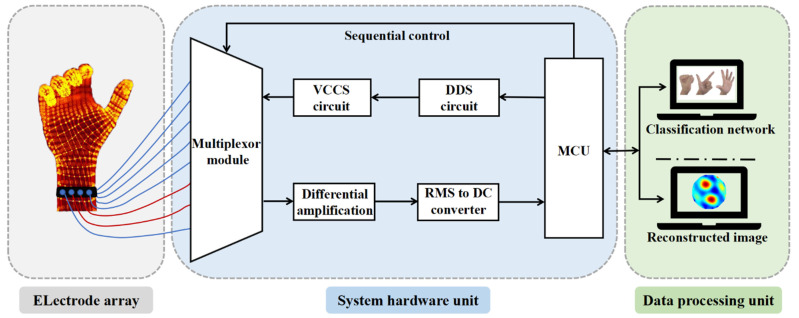
The overall structures of the electrical impedance tomography (EIT) system for gesture classification.

The entire EIT system architecture is shown in Figure 1. It consists of three parts: a data acquisition unit, system hardware unit, and data processing unit. The operation process of the gesture recognition system is roughly as follows. First, the optimal excitation measurement pattern is selected according to different measurement tasks. Then, the impedance data of the arm under different gestures is measured by the data acquisition system. Finally, the collected gesture data are sent to the computer for further data processing and hand gesture recognition.

**Figure 2:**
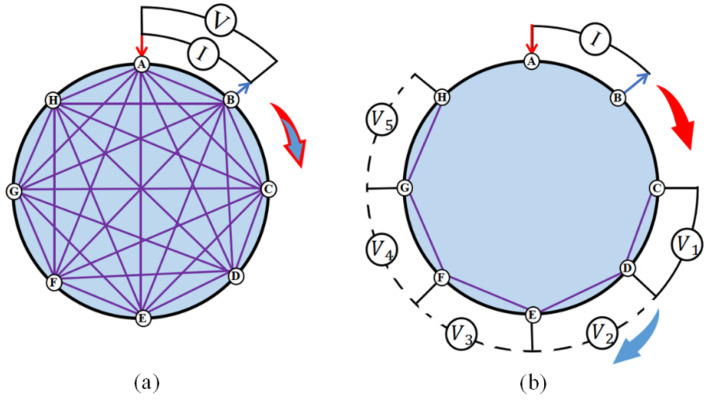
Two drive patterns. (**a**) Two-terminal scheme with eight electrodes. (**b**) Four-terminal scheme with eight electrodes. In the figure, the I represents the location of the injected excitation, and V represents the location of the voltage measurement. The red and blue arrows represent the direction of movement of the excitation and measurement electrodes, respectively.

The data acquisition mode used by most of EIT systems is cyclic excitation and measurement. Figure 2 shows the two most prevalent measurement strategies for cyclic excitation and measurement, namely two-terminal and four-terminal schemes [28,29]. For the two-terminal scheme, only one pair of electrodes is selected to capture impedance measurements. The selected electrode pair is multiplexed as the excitation electrode and the measurement electrode at the same time to complete the impedance measurement. However, the contact impedance produced by this method affects the imaging performance and classification accuracy. Today, the four-terminal schemes are widely used. In the four-terminal scheme, two pairs of electrodes are chosen to capture impedance measurements. Current is injected through one pair of electrodes, and the induced voltage is measured through the other pair.

As opposed to static gesture recognition, the impedance distribution of the arm changes quickly for dynamic gestures. The simultaneous variation of excitation current and impedance distribution for traditional cyclic excitation and measurement pattern introduces uncertain information and affects the recognition results. In order to keep the consistency of the same dynamic gesture data as much as possible, the fixed excitation pattern is adopted, which can simplify the measurement process to suit the impedance changes of dynamic gestures.

**Figure 3:**
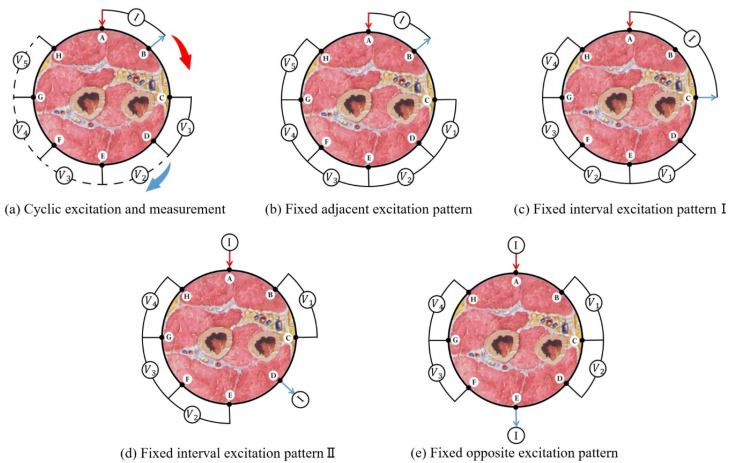
Different excitation drive patterns. The red and blue arrows represent the direction of movement of the excitation and measurement electrodes, respectively.

We discuss four fixed excitation patterns for the eight-electrode EIT system and compare them with the traditional cyclic excitation and measurement pattern, as shown in Figure 3. Figure 3a is traditional cyclic excitation and measurement pattern. Figure 3b–e are the fixed excitation measurement patterns used in this paper. According to the different positions of the excitation electrodes, it can be divided into a fixed adjacent excitation pattern, fixed interval excitation pattern, and fixed opposite excitation pattern. The measurement process of the fixed excitation pattern is as follows: during the gesture change process, a current signal is continuously applied to the selected excitation electrode pair, and then the resulting voltages between adjacent passive electrode pairs are measured cyclically.

### 2.2. System Architecture

**Figure 4:**
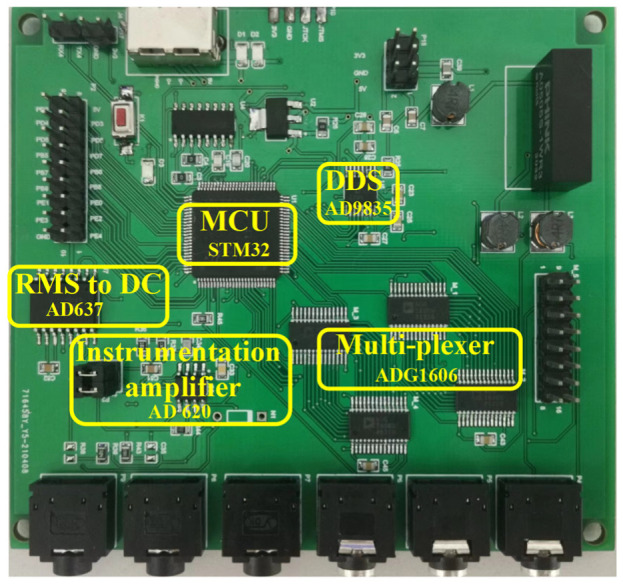
The data acquisition board.

**Figure 5:**
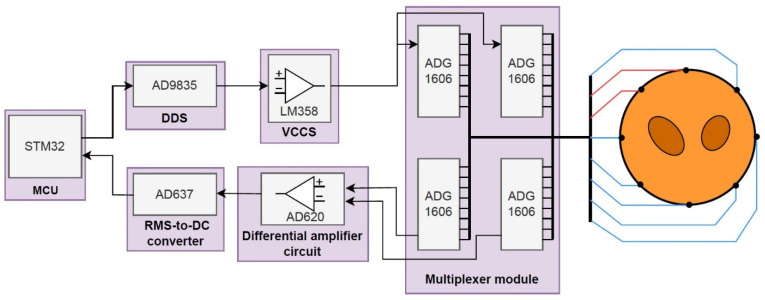
Overall architecture diagram of system hardware. The red line in the figure represents the input terminal of the excitation current, and the blue line represents the measurement terminal of the induced voltage.

The data acquisition board is shown in Figure 4. The overall architecture of the data acquisition system is shown in Figure 5. We use AD9835 DDS integrated circuit and an LM358 based Voltage Controlled Current Source (VCCS) to generate the EIT excitation signal. The AD9835 is configured to output a 30 kHz sine wave, which is fed into the VCCS for a constant 300µA AC output. We use four 16-to-1 multiplexers (ADG1606) as the multiplexer module. Two of the multiplexers connect the output terminal of the VCCS with excitation electrodes, and the other two multiplexers connect the buffer terminals of the instrumentation amplifier chip (AD620) with the measurement electrodes for voltage measurement. The 16-electrode scheme and the 8-electrode scheme can be switch freely through programming. The analogue to digital converter (ADC) module of the system is implemented by an effective-value direct current converter (AD637). The AD637 is a complete, high accuracy, root mean square (RMS) to direct current (DC) converter that computes the true RMS value of any complex wave form. The only external component required is a capacitor that sets the averaging period. The value of this capacitor also determines low frequency accuracy, ripple level, and settling time. The effective value of the signal is sampled through the AD port of the microcontroller unit (MCU) and stored in the buffer area.

For each dynamic gesture, 40 measured values can be obtained through measurement. The details of data acquisition for each measurement pattern are shown in Table 1. The contact between the electrode and the skin will introduce contact impedance, which brings inevitable errors to the collection of gesture data. This experiment uses a silver chloride medical ECG electrode with a conductive gel to reduce the contact impedance between the skin and the electrode in order to obtain stable and accurate measurement results.

### 2.3. Data Acquisition and Processing

**Figure 6:**
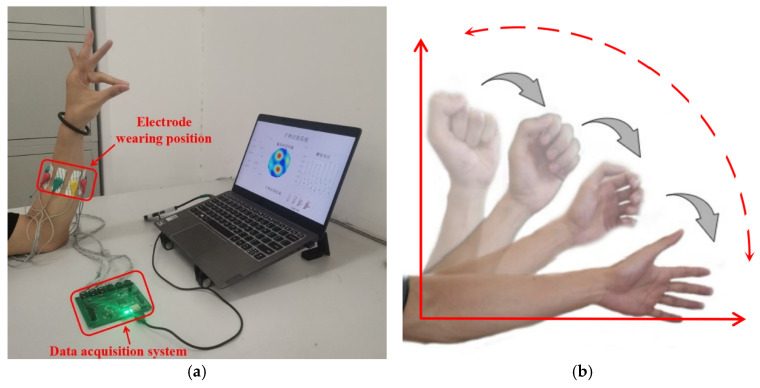
(**a**) Data acquisition experimental setup. (**b**) The shaking arm interference experiment.

**Figure 7:**
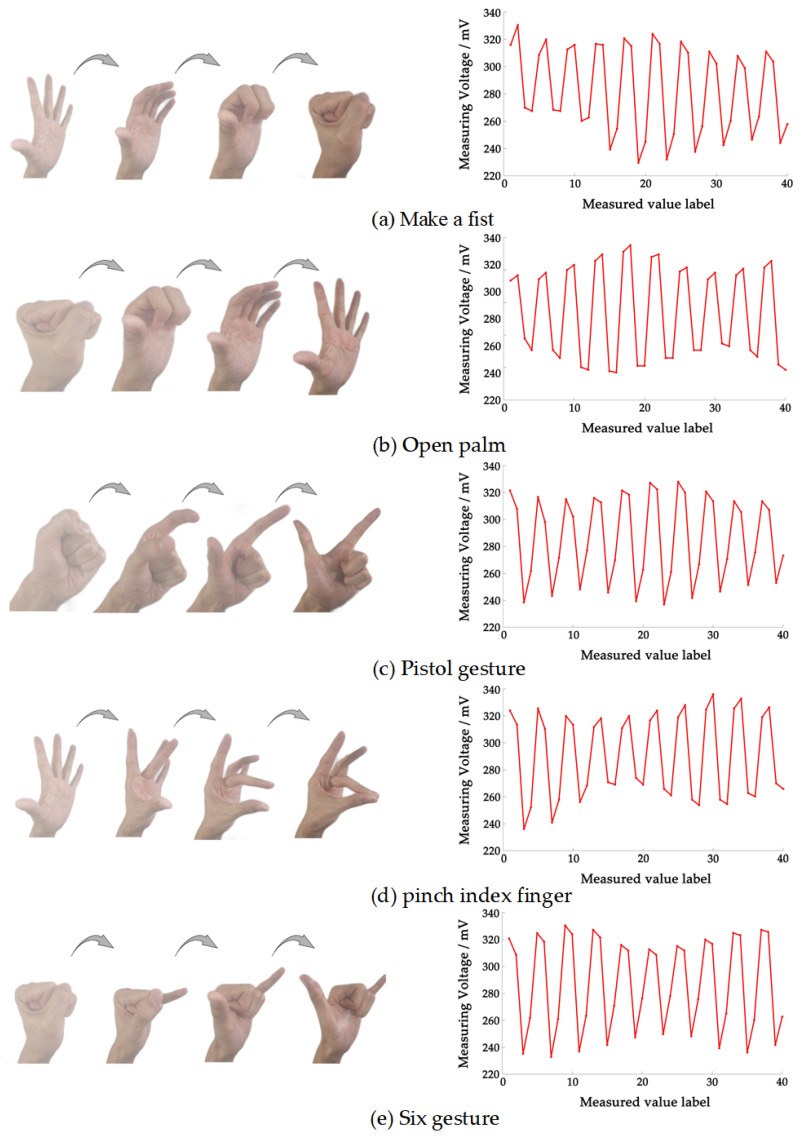
The dynamic gesture set and the measurement results of different gestures. The red solid line represents a measured data sequence for one dynamic gesture, which is composed of 40 measured voltages. The abscissa is the index of measured data, and the ordinate represents the amplitude of the measured voltage.

We recruited five healthy volunteers to participate in the experiment. The volunteers included 2 women and 3 men, all aged from 22 to 25 years old. Data acquisition can be achieved by volunteers completing hand gestures within the measurement time. In all experiments considered in this study, the left arm was used as the measurement object, and the electrodes were uniformly worn ten centimeters below the wrist, as shown in Figure 6a. Volunteers were asked to wash their arms with disinfectant alcohol and purified water before data acquisition. During data acquisition, volunteers were asked to put their arms on the table to keep them stable. We selected five dynamic gestures that are most commonly used and easy for volunteers to imitate and learn as classification objects, namely make a fist, open palm, pistol gesture, pinch index finger gesture, and “six” gestures. At the beginning of the experiment, the volunteers imitated the dynamic hand gestures displayed on the screen according to the prompts. After collecting 40 sets of hand gesture data, the volunteers were prompted to imitate a new hand gesture. This process was repeated until the volunteers completed data collection for the five hand gestures. This indicated that one iteration was completed. Volunteers were advised to rest for five minutes before each new gesture to prevent arm muscle fatigue. Each volunteer needs to go through four iterations, namely a session, without taking down or moving the device. The process resulted in 4000 sets of data (5 participants × 5 gestures × 40 sets of data × 4 iterations). The dynamic gesture set and the measurement results of different gestures are shown in Figure 7. 

In addition to collecting the normal measurement data, we also designed three kinds of interference experiments (i.e., shaking the arm, moving electrodes, and cross-day data classification) to verify the anti-interference ability of the network. In the shaking arm interference experiment, the initial position of the arm is perpendicular to the desktop. After the action starts, the arm begins to swing downward until it is horizontal to the desktop. During this process, the collection of gesture data is completed as shown in Figure 6b. This process is used to simulate the state of the arm during daily movements. Moving the electrode interference requires removing the measurement electrodes after each session and re-wearing them to start a new measurement after changing the electrode position. This process is used to generate deviations from different electrode positions. In the shaking arm and the moving electrodes interference experiments, the data acquisition process needs to be completed in a continuous time period. For each volunteer, the arm impedance status is different every day. To verify the accuracy of gesture classification in different time periods, we conducted a cross-day data classification experiment. The cross-day data collection requires each volunteer to collect data for three consecutive days. Data collection was performed at the same time period every day. To reduce cross-day data variance, we took care to place the EIT device with its electrodes in the same position relative to the subjects’ arms when data were acquired or when switching volunteers. While this reduces cross-day variance due to a rotation of the wristband, there are other sources of variance that are not easily controllable, namely: subtle deviations in electrode placement, changes in arm impedance, etc.

After the data collection was complete, we split it into two parts (one for training and the other for testing). The data set information is shown in Table 2. In the normal measurement data and the shaking arm interference data, the data collected in the first three iterations were used as the training set, and the data collected at the fourth iteration was used as the test set. In the moving electrode interference experiment, gesture data for six different electrode positions were collected, the first five iterations were used as the training set, and the last iteration was used as the test set. In the splitting of cross-day data, the data from the first two days were used to train the classification network, and the data from the third day were used to test the classification network.

## 3. Network Construction

### 3.1. Method

**Figure 8:**
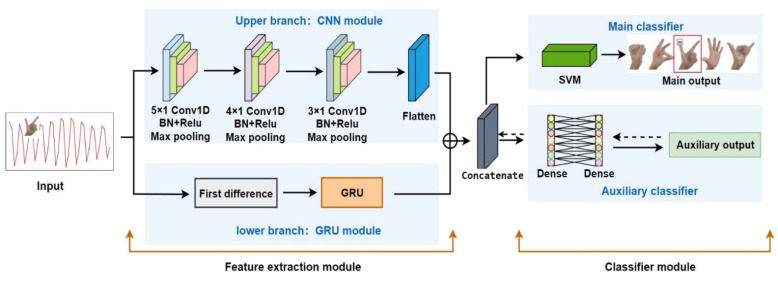
CG-SVM network structure.

In order to improve the generalization of the classification network, the network structure is redesigned in this paper. A dual-channel feature extraction network based on CNNs and Gated Recurrent Unit (GRU) [30] is proposed (i.e., the CG-SVM network). This classification network consists of two parts: feature extraction module and classifier module. As shown in Figure 8. The feature extraction module has upper (CNNs) and lower (GRU) branches. The output feature vector from feature extraction module are fed into the classifier for classification.

For the upper branch: the input data is sequentially fed into three convolutional neural network modules. The methods of CNNs processing time series can be divided into two types. First, the original one-dimensional data is rearranged into the form of a matrix, and then a two-dimensional convolutional network (2D-CNN) is used for feature extraction, which is suitable for the case that the original data has many eigenvalues. Second, a one-dimensional convolutional network (1D-CNN) is used to directly process one-dimensional data, which is suitable for the case that the original data has few eigenvalues. Each set of gesture data collected by this system contains only 40 measurement values. Due to the limitation of data size, one-dimensional convolution is used to convolve the data. To fully extract deep features from original data, multiple convolutional filters of size 5 × 1, 4 × 1, and 3 × 1 are used in the CNN module to learn features at different scales. In addition, the CNN module introduces sparsity through the ReLU function so as to better extract features and fit training data.

For the lower branch, the input measurement data is preprocessed by first-order difference. Then, multiple GRU units are utilized to extract gesture sequence features. GRU is a recurrent neural network that has been widely used for time series forecasting and classification [31,32]. The time series of EIT measurements records variation of muscle impedance during dynamic gestures, which is the input of the GRU network. Each GRU unit contains two control gates (a reset gate and an update gate). The reset gate combines the new input with the previous gesture feature information. The update gate selectively retains feature information and passes backwards. As a result, the output of each GRU unit comprehensively considers the current input and previous feature information, and fully excavates the interdependence between gesture sequence data.

In addition, the classifier module also contains two channels: the main classifier and the auxiliary classifier. SVM is used as the main classifier. The auxiliary classifier consists of two Dense layers. The feature vector of the feature extraction module is further passed down to the Dense layer, which is a regular fully connected layer and finally, it is forwarded to the classifier. The role of the auxiliary classifier branch is to adjust the eigenvalue distribution of the Concatenate layer through backpropagation, make it easier to be distinguished by SVM, and improve the classification accuracy.

### 3.2. Loss Function

SoftMax loss can make all of the classes have the maximum log-likelihood in the probability space, and is widely used in various classification networks as a loss function [33]. When SoftMax is used as the loss function, the deep features learned by the classification network will divide the entire hyperspace or hypersphere according to the number of categories to ensure that the categories are separable, but SoftMax does not require intra-class compactness and inter-class separation. Thus, there is a tricky problem. Although the deep features extracted by classification networks are more comprehensive, they do not necessarily have clustering characteristics. They will try to cover the entire space, which will have a negative impact on the judgment of the subsequent classifiers.

In this paper, a new loss function is proposed to improve the recognition accuracy of dynamic gestures. For dynamic gesture recognition, some different gestures have similar frames on the motion trajectory, which easily leads to misjudgment between them [34]. The inter-class spacing and intra-class compactness of different gesture features can be simultaneously supervised by our designed center distance loss, which can improve the discriminability between different dynamic gestures. The complete loss function is shown in Equation (1):

(1) $$L\;=L_{s}+\lambda_{1}L_{c}+\lambda_{2}L_{d}$$

$L_{s}$ is the Softmax loss function, as shown in Equation (2). $x_{i}\in R^{d}$ denotes the ith deep feature, belonging to the $y_{i}$th class. d is the feature dimension. $W_{j}\in R^{d}$ denotes the jth column of the weights $W\in R^{d\times n}$ in the last fully connected layer and $b\in R^{n}$ is the bias term. The size of mini-batch and the number of class is m and n, respectively.

(2) $$\;L_{s}=-\overset{m}{\underset{i=1}{\sum }}\log\frac{e^{W_{y_{i}}^{T}x_{i}+b_{y_{i}}}}{\sum_{j=1}^{n}e^{W_{j}^{T}x_{i}+b_{j}}}$$

(3) $$\;L_{c}=\frac{1}{2}\overset{m}{\underset{i=1}{\sum }}\|x_{i}-c_{y_{i}}\|{}_{2}^{2}$$

$L_{c}$ defined in Equation (3) is the Center loss supervision item [35], $c_{y_{i}}\in R^{d}$ denotes the $y_{i}$th class center of deep features.

The loss function combining $L_{s}$ and $L_{c}$ can better achieve the learning objective of intra-class compactness, but it lacks the control of inter-class distance. To supervise the inter-class spacing of different gesture features, we design a center distance penalty item $L_{d}$ as shown in Equation (4).

(4) $$\;\;\;\;\;\;\;L_{d}=\overset{m}{\underset{i=1}{\sum }}\max\left(0,K-\|c_{y_{i}}-c_{{\overset{ˇ}{y}}_{i}}\|{}_{2}^{2}\right)$$

The $c_{{\overset{ˇ}{y}}_{i}}\in R^{d}$ denotes the class center of other deep features except $c_{y_{i}}$. *K* is a distance coefficient specified through experience. The inter-class spacing is supervised by calculating the distance between the centers of different classes, thereby improving the distinguishability of confusing gesture data. The purpose of adopting the max form in Equation (4) is to improve the discrimination of features with close center distances while maintaining distinguishable feature center distances.

In the whole loss function (Equation (1)), the balance of the center loss term and the center distance penalty term is adjusted by scalars $\lambda_{1}$ and $\lambda_{2}$.

## 4. Results and Discussion

### 4.1. Drive Mode Comparison

All types of gesture data were successfully collected through the five excitation drive patterns described in Figure 3. Three machine learning algorithms, namely decisiontTree, KNN and SVM, were used to classify the data. Statistical relevance was evaluated using a one-way analysis of variance (ANOVA) with a significance value of *p* < 0.05. Post hoc tests were performed using the Holm–Bonferroni correction if the variances of the average accuracy were homogeneous. Post hoc tests were performed using Tamhane T2 if the variances were heterogeneous.

**Figure 9:**
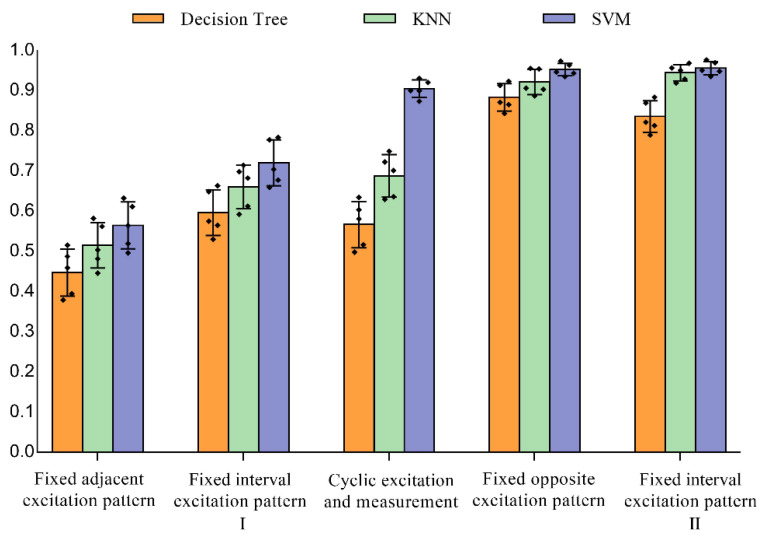
Classification results of normal measurement data under different excitation drive patterns, the vertical lines depict the respective 95% confidence intervals, the black squares in the figure represent the distribution of sample points.

Figure 9 shows the classification results of each algorithm on normal measurement data. The classification results of each algorithm were analyzed by one-way ANOVA. Among the four fixed excitation measurement patterns, the fixed interval excitation pattern Ⅱ and the opposite excitation pattern stand out, which are significantly better than the traditional cyclic excitation and measurement pattern (pairwise *p* < 0.041, d ≥ 4.7). This shows that our proposed fixed excitation measurement pattern achieves good results in dynamic gesture recognition. Comparing the performance of the three classification algorithms from the experimental results, the SVM algorithm achieves the best classification accuracy under the same excitation pattern.

**Figure 10:**
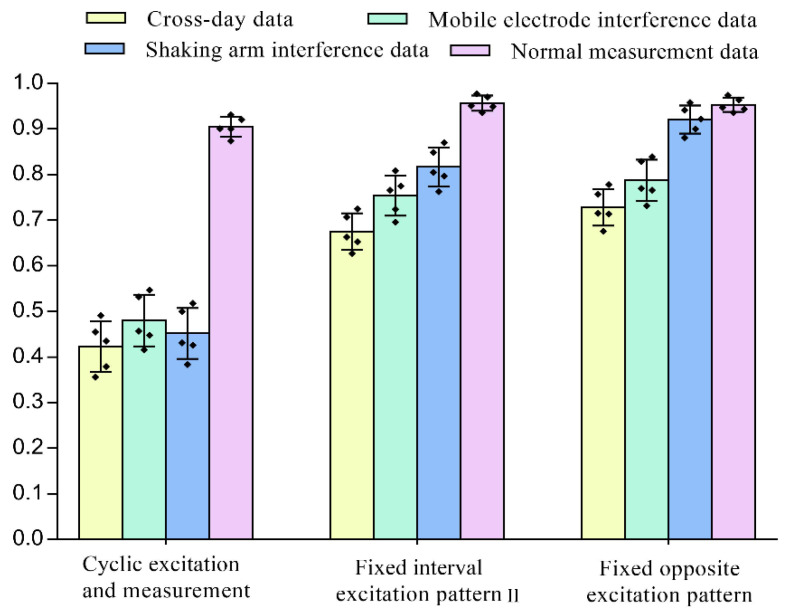
Classification results of normal data and interference data under different excitation patterns, the vertical lines depict the respective 95% confidence intervals, the black squares in the figure represent the distribution of sample points.

The classification results of normal data and interference data under different excitation patterns are shown in Figure 10. From the classification results of the SVM algorithm, the classification accuracy of the traditional cyclic excitation and measurement pattern dropped significantly in the three interference experiments. The classification accuracy of the other two fixed excitation patterns is also decreased, but was still significantly better than the cyclic excitation and measurement modalities (pairwise *p* < 0.001, d ≥ 25). This further reflects the advantages of the proposed fixed excitation measurement pattern in the interference environment. Compared with the fixed interval excitation patternⅡ, the fixed opposite excitation pattern performs better in the classification task of interference data, especially in the shaking arm interference test, the classification accuracy is significantly better than that of the fixed interval excitation patternⅡ (*p* < 0.009, d = 11.3).

**Figure 11:**
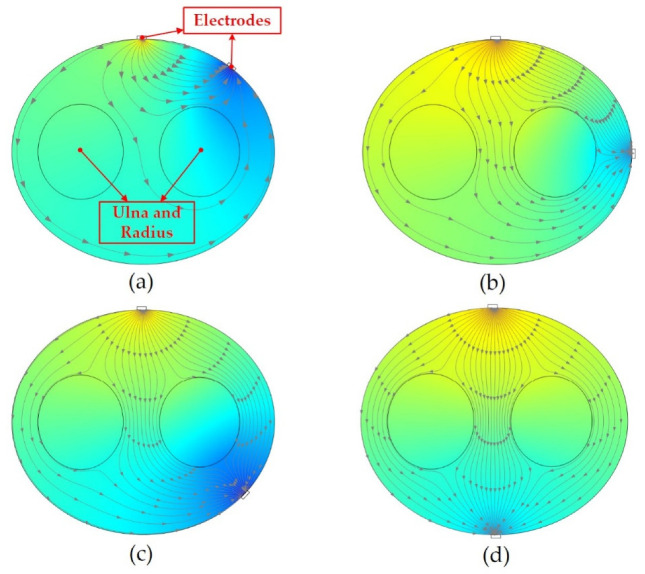
Simulation analysis of different excitation measurement patterns. The big ellipse represents the cross-section of our arm. The two small ellipses are the ulna and radius inside the arm, and the gap represents the arm muscle.

We conducted a simulation analysis to explain the advantages of fixed opposite ex-citation pattern in anti-interference experiments. The simulation result is shown in Figure 11. The excitation electrodes were applied with a current of 300 µA, which is the same with the real EIT system. The conductivity of the arm bone was set to 0.02043 S/m, and the conductivity of the arm muscle was set to 0.5448 S/m [36,37].

From the simulation results, the distance between the excitation electrodes for the fixed adjacent excitation pattern (Figure 11a) and the fixed interval excitation pattern Ⅰ (Figure 11b) is too close, the muscles on the other side of the arm are less stimulated by the current, the induced voltage is relatively weak. As a result, the recognition results are affected. From the simulation results of the fixed interval excitation pattern Ⅱ (Figure 11c) and the fixed opposite excitation pattern (Figure 11d), We can see that the electric field lines are distributed more uniformly and densely inside the arm, which is more sensitive to muscle impedance variation. Under the same conditions of current amplitude, the electric field lines distribution of the fixed opposite excitation pattern (Figure 11d) is the most uniform, so it has the strongest anti-interference ability.

In summary, we can draw the following conclusions: in the dynamic gesture recognition task, fixed excitation pattern has more advantages than the cyclic excitation pattern. The fixed interval excitation pattern Ⅱ achieved the best results under the normal measurement data, but the fixed opposite excitation pattern has stronger anti-interference ability.

### 4.2. Comparison of Classification Network Results

In order to evaluate the performance of proposed classification network, comparative experiments with different network and loss functions were conducted.

The detailed parameters of all networks used in the experiments are shown in Table 3. The network structure of CNNs is the same as the upper branch of the dual-channel feature extraction network proposed in this paper, which is used to verify the reliability of the new network structure. The CG-SVM3 is the new method proposed in this paper. It should be noted that CG-SVM1, CG-SVM2, and CG-SVM3 have the same network structure, but different loss functions are used by these networks. The loss function of CG-SVM1, CG-SVM2, SVM3 are SoftMax loss, center loss, and center distance loss, respectively. The comparison of these three networks can be used to verify the performance of the proposed center distance loss function.

**Figure 12:**
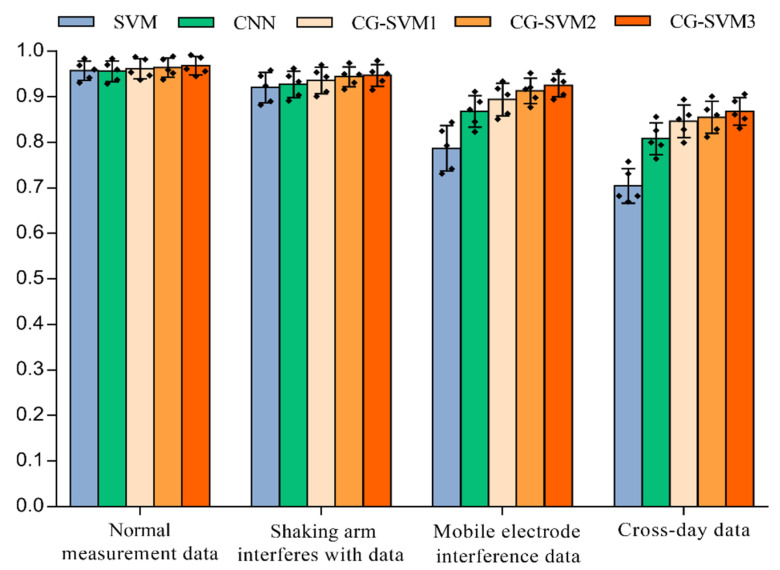
Comparison of network classification accuracy, the vertical lines depict the respective 95% confidence intervals, the black squares in the figure represent the distribution of sample points.

The recognition results of different types of data under different networks are shown in Figure 12. Compared with the SVM algorithm and the CNN network, the recognition accuracy of the CG-SVM3 network is dramatically improved, especially for interfered data. In the moving electrode interference experiment, the classification accuracy of CG-SVM3 network is 92.5%, which is 13.8% and 5.7% higher than SVM algorithm and CNN network respectively (*p* < 0.006, d = 13.8 and *p* < 0.05, d = 5.7). In the classification task of cross-day data, the classification accuracy of CG-SVM3 network is 14.2% and 6% higher than that of SVM algorithm and CNN network, respectively (*p* < 0.008, d = 14.2 and *p* < 0.05, d = 6). This further confirms the excellent feature extraction ability and anti-interference ability of the newly designed classification network.

**Figure 13:**
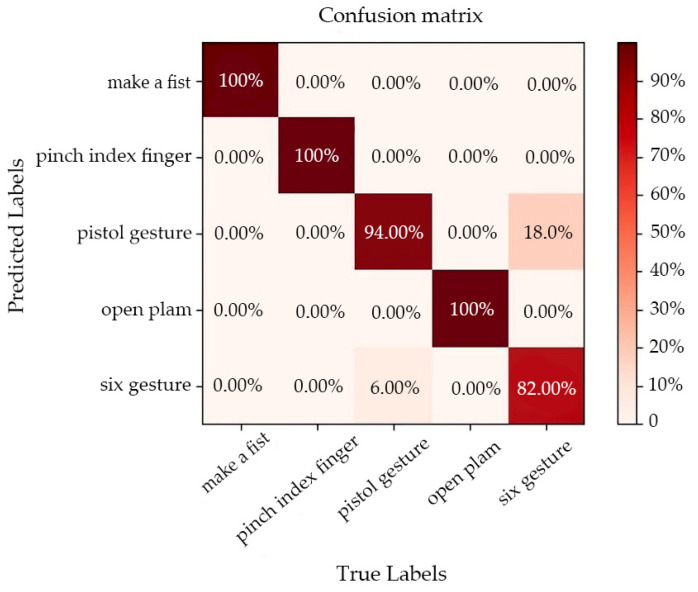
Confusion matrix of normal measurement data classification results.

**Figure 14:**
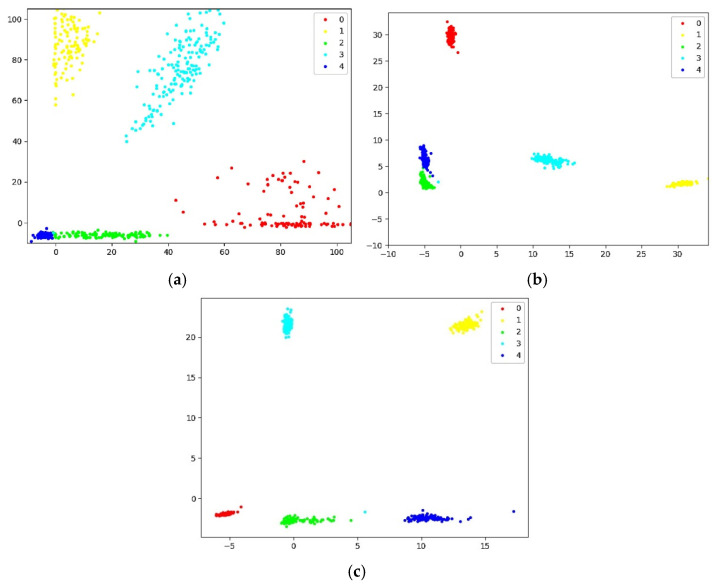
The output feature map of the network under different loss functions: (**a**) SoftMax loss; (**b**) center loss; (**c**) center distance loss. The points with different colors denote features from different classes.

Figure 13 is a confusion matrix of the classification results of the normal measurement data. From the confusion matrix, we can find that the recognition errors mainly occur in the ‘six’ hand gesture and the ‘pistol’ hand gesture. A total of 18% of the ‘six’ hand gestures are incorrectly identified as the ‘pistol’ hand gestures, and 6% of the ‘pistol’ hand gestures are incorrectly recognized as the ‘six’ hand gesture. Figure 14 is the output feature map of the network under different loss functions. We can see that in the feature map of SoftMax loss (Figure 14a), the feature points of the same category are scattered in space, and some of the feature points of gesture two (‘pistol ‘ hand gesture) and gesture four (‘six’ hand gesture) blend together, lacking class spacing, so the recognition error rate of these two hand gestures is the highest. When center loss was used as the loss function, it can calculate the distance between the feature point of each category and its feature center, and can continuously reduce this distance. Therefore, the distribution of the features of the same category in the space will be more concentrated, as shown in Figure 14b. However, center loss only considers the intra-class distance and lacks the supervision of the inter-class distance. The proposed center distance penalty item can make up for this deficiency. It not only considers the intra-class distance of the same type of feature points, but also supervises and punishes the inter-class distance of different types of feature points. As shown in Figure 14c, the feature points of each category have achieved intra-class compactness and increased the inter-class spacing, so the CG-SVM3 network achieves a higher recognition accuracy.

### 4.3. Limitations and Outlooks

For the fixed excitation mode proposed in this paper, only electrodes with fixed positions (electrode A and E in Figure 3e of revised manuscript.) are used for current injection. However, the characteristics of conductivity distribution for different gestures need to be discussed, so that the fixed current excitation electrodes could be positioned in sensitive area according to different gestures. Thereby, the best measurement performance could be obtained.

The whole body movement recognition based on EIT is still a research trend, although there are some challenges. Since the electrode sensor of current EIT system cannot cover the entire body, wearable sensor adapted to different parts of the human body should be designed. The impedance distribution corresponding to the different postures needs to be understood, and the data stability for simultaneous measurement of different body parts needs to be improved. More efficient recognition network with high accuracy will also be studied. Therefore, more research and experiments are needed to verify this in future.

## 5. Conclusions

We propose an optimal excitation driven pattern for EIT dynamic gesture measurement. We also proposed a dual-channel feature extraction network CG-SVM to extract deep features from gesture data. To solve the problem that some dynamic hand gesture features are not highly distinguishable, the center distance loss is proposed to increase the inter-class spacing as much as possible, so that the distinguishability of various dynamic gestures has been improved. The new network structure and loss function proposed in this paper have achieved higher recognition effect and stronger anti-interference ability.

## Figures and Tables

**Table 1:** Data collection details for different measurement methods.

Excitation Pattern	Excitation Electrode Pair	Measuring Electrode Pair	Number of Cycles	Data Volume
Fixed adjacent excitation pattern	A, B	C, D; D, E; E, F; F, G; G, H	8	40
Fixed interval excitation pattern Ⅰ	A, C	D, E; E, F; F, G; G, H	10	40
Fixed interval excitation pattern Ⅱ	A, D	B, C; E, F; F, G; G, H	10	40
Fixed opposite excitation pattern	A, E	B, C; C, D; F, G; G, H	10	40

**Table 2:** Dataset details.

Type of Data		Training Set (Quantity)	Test Set (Quantity)
1	Normal measurement data	The 1st, 2nd, 3rd iteration data (3000)	The 4th iteration data (1000)
2	Shaking arm interferes with data	The 1st, 2nd, 3rd iteration data (3000)	The 4th iteration data (1000)
3	Moving electrode interference data (Cross-session data for same time periods)	The 1st, 2nd, 3rd, 4th, 5th iteration data (5000)	The 6th iteration data (1000)
4	Cross-day data (Cross-session data for different time periods)	The 1st and 2nd day data (2000)	The 3rd day data (1250)

**Table 3:** Detailed parameters of each network model.

	CNNs	CG-SVM1		CG-SVM2		CG-SVM3	
Conv1_x	[5 × 1, 16]	[5 × 1, 16]	GRU	[5 × 1, 16]	GRU	[5 × 1, 16]	GRU
Conv2_x	[4 × 1, 32]	[4 × 1, 32]		[4 × 1, 32]		[4 × 1, 32]	
Conv3_x	[3 × 1, 64]	[3 × 1, 64]		[3 × 1, 64]		[3 × 1, 64]	
Dropout	0.5	0.5		0.5		0.5	
Classifier	Softmax	SVM		SVM		SVM	
Loss function	Softmax loss	Softmax loss		Center loss		Center distance loss	

## Data Availability

The data presented in this study are available on request from the corresponding author.
